# Pulmonary function, chronic respiratory symptoms, and health-related quality of life among adults in the United States – National Health and Nutrition Examination Survey 2007–2010

**DOI:** 10.1186/1471-2458-13-854

**Published:** 2013-09-17

**Authors:** Anne G Wheaton, Earl S Ford, William W Thompson, Kurt J Greenlund, Letitia R Presley-Cantrell, Janet B Croft

**Affiliations:** 1 Division of Population Health, National Center for Chronic Disease Prevention and Health Promotion, Centers for Disease Control and Prevention, 4770 Buford Highway, Mailstop K67, Atlanta, GA, 30341, USA

**Keywords:** Chronic respiratory disease, Spirometry, FEV1, Activity limitation, Frequent mental distress, Wellbeing

## Abstract

**Background:**

We examined the association of impaired lung function and respiratory symptoms with measures of health status and health-related quality of life (HRQOL) among US adults.

**Methods:**

The sample included 5139 participants aged 40–79 years in the National Health and Nutrition Examination Survey 2007–2010 who underwent spirometric testing and responded to questions about respiratory symptoms, health status, and number of physically unhealthy, mentally unhealthy, or activity limitation days in the prior 30 days.

**Results:**

Among these adults, 7.2% had restrictive impairment (FEV_1_/FVC ≥ 70%; FVC < 80% of predicted), 10.9% had mild obstruction (FEV_1_/FVC < 70%; FEV_1_ ≥ 80% predicted), and 9.0% had moderate–severe obstruction (FEV_1_/FVC < 70%; FEV_1_ < 80% predicted). Individuals with restrictive impairment or moderate–severe obstruction were more likely to report fair/poor health compared to those with normal lung function (prevalence ratio (PR) =1.5 [95% CI: 1.2-1.9] and 1.5 [1.3-1.8]), after controlling for sociodemographics, non-respiratory chronic diseases, body mass index, smoking, and respiratory symptoms. Frequent mental distress (FMD; ≥14 mentally unhealthy days), frequent physical distress (FPD; ≥14 physically unhealthy days), and frequent activity limitation (FAL; ≥14 activity limitation days) did not differ by lung function status. Adults who reported any respiratory symptoms (frequent cough, frequent phlegm, or past year wheeze) were more likely to report fair/poor health (PR = 1.5 [1.3-1.7]), FPD (PR = 1.6 [1.4-1.9]), FMD (PR = 1.8 [1.4-2.2]), and FAL (PR = 1.4 [1.1-1.9]) than those with no symptoms.

**Conclusions:**

These results suggest the importance of chronic respiratory symptoms as potential risk factors for poor HRQOL and suggest improved symptom treatment and prevention efforts would likely improve HRQOL.

## Background

Impaired pulmonary function is common in the United States and is often undiagnosed [1]. Approximately 14% of US adults have some obstructive impairment of lung function, primarily chronic obstructive pulmonary disease (COPD) and asthma while another 6% have restrictive impairment, a characteristic of many interstitial pulmonary diseases [2]. Poor health-related quality of life (HRQOL) is common among patients diagnosed with COPD, asthma, and other chronic respiratory diseases [3-8]. As with other chronic illnesses, a principal goal in the management of these conditions is to improve the quality of life of the individuals suffering from these respiratory conditions. In order to implement strategies to improve HRQOL, it is important to recognize the important determinants of poor HRQOL. Two potential contributors to poor HRQOL in respiratory diseases are impaired lung function, as assessed by spirometry, and respiratory symptoms such as frequent cough, phlegm production, or wheeze. Although pulmonary function impairment has been found to be associated with poor HRQOL in some studies, most studies have been conducted among small, clinical populations and not the general population [9-12]. In addition, most research has not included data on respiratory symptoms, relying primarily on spirometric measures of disease severity [10]. Also, most studies have not evaluated the association between respiratory symptoms and HRQOL septely from pulmonary function; therefore they have not been able to assess whether poor HRQOL was attributable to impaired pulmonary function rather than respiratory symptoms.

The objective of this study was to examine the independent associations of impaired lung function and respiratory symptoms with measures of health status and HRQOL among US adults.

## Methods

### Study population

National Health and Nutrition Examination Survey (NHANES) is a stratified, multistage probability sample of the civilian non-institutionalized US population. The survey is conducted by the National Center for Health Statistics, Centers for Disease Control and Prevention. Additional information is available at http://www.cdc.gov/nchs/nhanes.htm. Participants are interviewed in their homes and most subsequently complete health examination components that include medical examinations, usually administered in mobile examination centers (MEC). Low-income persons, persons aged ≥60 years, Hispanics, and non-Hispanic blacks were oversampled in 2007–2010. Many of the interviewers are bilingual (English-Spanish), but efforts are made to obtain interpreters for the small minority of participants who communicate in languages other than English and Spanish. Because the respiratory symptom questions were asked only of adults aged ≥40 years and spirometry was not performed on adults aged ≥80 years, the sample for this study was limited to adults aged 40–79 years who had completed baseline spirometry and responded to questions regarding respiratory symptoms in 2007–2010. NHANES was approved by the Research Ethics Review Board of the National Center for Health Statistics.

### Pulmonary function

Pulmonary function was assessed by spirometry at the MEC on eligible participants aged 6–79 years [13]. Examinees who reported current chest pain or had a physical problem that may have prevented them from forceful expiration were excluded, as were those who were using supplemental oxygen; reported recent eye, chest, or abdominal surgery; had a recent heart attack, stroke, or tuberculosis exposure; had recently coughed up blood; or reported a history of detached retina or collapsed lung. Exams with questionable or invalid results were also excluded. More details regarding spirometry quality are also discussed in the NHANES documentation [13]. Variables obtained from spirometry and used in this study included forced expiratory volume in 1 second (FEV_1_), forced vital capacity (FVC), and the FEV_1_/FVC ratio. Predicted values of FEV_1_ and FVC were calculated using previously published equations [14] and data on participants’ age, sex, height, and race/ethnicity. The participants were classified into the following categories: normal (FEV_1_/FVC ≥ 70%; FVC ≥ 80% predicted), mild obstruction (FEV_1_/FVC < 70%; FEV_1_ ≥ 80% predicted), moderate–severe obstruction (FEV_1_/FVC < 70%; FEV_1_ <80% predicted), or restricted (FEV_1_/FVC ≥ 70%; FVC < 80% predicted).

### Chronic respiratory symptoms

During the home interview, participants aged ≥40 years were asked if they “usually cough on most days for 3 consecutive months or more during the year”, “bring up phlegm on most days for 3 consecutive months or more during the year”, or “had wheezing or whistling in their chest” in the past 12 months.

### Health-related quality of life (HRQOL)

The current health status questionnaire, which included questions on overall health status and general HRQOL, was administered during the physical examination at the MEC. Fair/poor health was defined as a response of fair or poor to the question, “Would you say your health in general is excellent, very good, good, fair, or poor?” Participants were also asked, “Thinking about your physical health, which includes physical illness and injury, for how many days during the past 30 days was your physical health not good?”; “Now thinking about your mental health, which includes stress, depression, and problems with emotions, for how many days during the past 30 days was your mental health not good?”; and “During the past 30 days, for about how many days did poor physical or mental health keep you from doing your usual activities, such as self-care, work, school or recreation?” The construct validity of these four HRQOL questions has been discussed previously [15,16]. A response of ≥14 days to each question was categorized as frequent physical distress, frequent mental distress, and frequent activity limitation, respectively. The 14-day cutoff has been used in prior studies using these Healthy Days measures and reflects the upper 10-15% of the distribution for each of these measures [17,18].

### Covariates

Data were also collected regarding participants’ sex, age, race/ethnicity (non-Hispanic white, non-Hispanic black, Mexican-American, and other), and years of education (<12 years, 12 years, >12 years). Participants were also asked about chronic non-respiratory illnesses including diabetes, arthritis, gout, current anemia, heart failure, coronary heart disease, angina, myocardial infarction, stroke, thyroid disease, liver disease, and any cancer, and were categorized based on number of chronic illnesses (0, 1–2, ≥3). Participants were also categorized by smoking status (current, former, never). Participant height and weight were measured by trained health technicians and used to calculate body mass index (BMI) (weight [kg]/height [m^2^]).

### Statistical analysis

Statistical analyses were conducted using SAS 9.2 (SAS Institute, Inc.) with SUDAAN 10.0.1 (RTI International) to account for the complex sampling design. MEC sample weights were used. The proportion of participants with respiratory symptoms and prevalence ratios (PR) with 95% confidence intervals (CI) for the likelihood of respiratory symptoms associated with lung function status were obtained from multivariable logistic regression analyses that were adjusted for sex, age, race/ethnicity, education level, number of non-respiratory chronic illnesses, BMI, and smoking status. The proportion of participants with poor HRQOL measures and prevalence ratios (PR) with 95% confidence intervals (CI) for the likelihood of poor HRQOL associated with lung function status and respiratory symptoms were obtained from multivariable logistic regression analyses that also adjusted for sex, age, race/ethnicity, education level, number of non-respiratory chronic illnesses, BMI, and smoking status. We investigated the effects of lung function status and respiratory symptoms both septely (models included either lung function category or respiratory symptoms) and simultaneously (models included both lung function category and respiratory symptoms). Respiratory symptoms were assessed individually (frequent cough, frequent phlegm, or past year wheeze) and as a binary variable (any vs. none).

## Results

Out of 7296 adults aged 40–79 years who participated in the 2007–2010 NHANES cycle, 192 did not undergo examinations at the MECs, 1643 were excluded due to inadequate spirometry data (safety exclusions = 649 (Table 1), unacceptable quality spirometry = 346, and did not undergo testing for other reasons = 648), 314 were missing at least one response to the HRQOL or respiratory symptoms questions, and 8 were excluded due to missing education or BMI data. The final study sample consisted of 5139 individuals (70.4% of the initial sample).

**Table 1:** Participants excluded from having spirometry for safety-related reasons (N = 649)

**Reason for exclusion**	**n**
Current breathing problem requiring supplemental oxygen	101
Current pain or physical problem preventing taking a deep breath	150
Coughed up blood in previous month	17
Eye surgery in previous 3 months	74
Open chest or abdominal surgery in previous 3 months	42
Stroke in previous 3 months	9
Heart attack in previous 3 months	7
Tuberculosis in previous year	21
History of aneurysm	66
History of collapsed lung	79
History of detached retina	83

Characteristics of the sample population are presented in Table 2. Of 5139 participants, 2572 (49.0%) were men, 2231 (32.1%) were aged 60–79 years, 2566 (75.4%) were white, and 2447 (57.7%) had >12 years education. In addition, 9.4% reported ≥3 non-respiratory chronic illnesses, 37.3% were obese (BMI ≥ 30 kg/m^2^), and 18.4% were current smokers. Lung function was normal for 72.9% while 7.2% were categorized as having restrictive impairment and 19.9% had at least mild obstruction. The most common respiratory symptom was wheezing in the past year (12.9%) and 21.0% reported at least one of the three respiratory symptoms studied. Among the participants who reported respiratory symptoms, 64% reported only one symptom and 12% reported all three. Fair/poor health was reported by 16.3%. Frequent physical distress, mental distress, and activity limitation were reported by 11.3%, 11.6%, and 5.3% of the study population. Among participants who reported fair/poor health, 32% also reported frequent physical distress, 25% reported frequent mental distress, and 18% reported frequent activity limitation. Of those with frequent physical distress, less than half rated their health as fair or poor (47%), with corresponding proportions of 35% for those with frequent mental distress and 56% for those with frequent activity limitations.

**Table 2:** Unadjusted characteristics of study population - NHANES, 2007–2010

**Characteristic**	**N**^**a**^	**%**^**b**^	**(95% CI)**
**Total**	5139	100.0	
**Sex**			
Men	2572	49.0	(47.8-50.1)
Women	2567	51.0	(49.9-52.2)
**Age category (years)**			
40-49	1523	35.2	(32.9-37.6)
50-59	1385	32.7	(30.8-34.6)
60-69	1364	21.0	(19.6-22.4)
70-79	867	11.1	(10.3-12.0)
**Race/Ethnicity**			
White (Non-Hispanic)	2566	75.4	(70.5-79.7)
Black (Non-Hispanic)	963	9.7	(7.7-12.1)
Mexican-American	872	6.2	(4.2-8.9)
Other/Multiracial	738	8.8	(6.8-11.3)
**Education (years)**			
<12	1467	17.4	(15.3-19.7)
12	1225	24.9	(22.7-27.3)
>12	2447	57.7	(54.1-61.3)
**Non-respiratory chronic illnesses**^**c**^			
None	2164	45.3	(42.4-48.2)
1-2	2395	45.3	(43.0-47.7)
≥3	580	9.4	(8.1-10.8)
**BMI category**			
Underweight/normal (<25.0 kg/m^2^)	1177	25.8	(23.6-28.2)
Overweight (25.0-29.9 kg/m^2^)	1885	36.9	(34.6-39.2)
Obese (≥30 kg/m^2^)	2077	37.3	(35.1-39.5)
**Smoking status**			
Current	1036	18.4	(16.5-20.6)
Former	1563	30.3	(28.0-32.7)
Never	2539	51.3	(48.8-53.8)
**Lung function category**			
Normal lung function	3698	72.9	(70.8-74.8)
Restrictive impairment	438	7.2	(6.2-8.4)
Mild obstruction	524	10.9	(9.4-12.6)
Moderate–severe obstruction	479	9.0	(7.8-10.3)
**Respiratory symptoms**			
Frequent cough	521	10.4	(9.0-12.0)
Frequent phlegm	445	7.8	(7.0-8.7)
Past year wheeze	678	12.9	(11.4-14.5)
Any symptoms	1087	21.0	(19.0-23.2)
No symptoms	4052	79.0	(76.8-81.0)
1 symptom	670	13.4	(11.9-15.2)
2 symptoms	277	5.0	(4.3-5.9)
3 symptoms	140	2.5	(2.0-3.2)
**Health-related quality of life**			
Fair/poor health	1248	16.3	(14.4-18.4)
Frequent physical distress^d^	691	11.3	(9.7-13.1)
Frequent mental distress^e^	627	11.6	(10.2-13.0)
Frequent activity limitation^f^	322	5.3	(4.4-6.4)

^a^ Unweighted n’s.

^b^ Weighted percentage.

^c^ Non-respiratory chronic diseases included diabetes, arthritis, gout, current anemia, heart failure, coronary heart disease, angina, myocardial infarction, stroke, thyroid disease, liver disease, and any cancer.

^d^ Physical health not good ≥14 days during past 30 days.

^e^ Mental health not good ≥14 days during past 30 days.

^f^ Poor health limited usual activities ≥14 days during past 30 days.

When compared to participants with normal lung function, those with moderate–severe obstruction were more likely to report frequent cough (PR = 1.63 [1.20-2.20]), frequent phlegm (PR = 1.99 [1.31-3.03]), and wheeze in the past year (PR = 2.65 [2.04-3.43]) (Table 3). Report of individual respiratory symptoms was not significantly elevated among participants with restrictive impairment or mild obstruction, although those with restrictive impairment were more likely to report at least one respiratory symptom (PR = 1.32 [1.06-1.63]). Less than half (47.4%) of participants with moderate–severe obstruction reported at least one of the respiratory symptoms.

**Table 3:** **Likelihood of respiratory symptoms**^**a **^**by lung function category**

	**%**^**b **^**(95% CI)**	**Adjusted PR**^**c **^**(95% CI)**	
**Frequent cough**			
Normal lung function	8.6 (7.1-10.3)	1.00	--
Restrictive impairment	13.0 (8.9-18.7)	1.12	(0.75-1.66)
Mild obstruction	9.3 (6.4-13.2)	0.83	(0.53-1.29)
Moderate–severe obstruction	24.2 (18.7-30.7)	1.63	(1.20-2.20)
**Frequent phlegm**			
Normal lung function	5.9 (5.2-6.8)	1.00	--
Restrictive impairment	10.0 (6.4-15.3)	1.14	(0.72-1.81)
Mild obstruction	9.1 (6.0-13.6)	1.21	(0.76-1.93)
Moderate–severe obstruction	19.8 (14.5-26.4)	1.99	(1.31-3.03)
**Wheeze**			
Normal lung function	10.2 (9.0-11.5)	1.00	--
Restrictive impairment	18.5 (13.7-24.6)	1.36	(0.99-1.87)
Mild obstruction	11.8 (8.7-15.7)	1.23	(0.89-1.71)
Moderate–severe obstruction	31.9 (25.8-38.8)	2.65	(2.04-3.43)
**Any respiratory symptoms**			
Normal lung function	17.1 (15.1-19.3)	1.00	--
Restrictive impairment	29.0 (23.8-34.8)	1.32	(1.06-1.63)
Mild obstruction	19.9 (15.8-24.8)	1.06	(0.81-1.38)
Moderate–severe obstruction	47.4 (39.9-55.0)	2.10	(1.69-2.60)

^a^ Respiratory symptoms included the following: cough most days (at least 3 months), bring up phlegm most days (at least 3 months), or had wheeze or whistle in chest (in past 12 months).

^b^ Unadjusted.

^c^ Adjusted prevalence ratio (PR) and 95% confidence interval (CI) obtained from multivariable logistic regression that included sex, age, race/ethnicity, education, number of non-respiratory chronic illnesses, smoking status, and BMI as covariates.

Compared to those with normal lung function, the unadjusted prevalences of all poor HRQOL measures were significantly higher (p < 0.05) for those with restrictive impairment (Table 4). With the exception of frequent mental distress, the prevalences of poor HRQOL measures also were significantly higher for participants with moderate–severe obstruction. Mild obstruction was not associated with any of the HRQOL measures. After adjusting for sociodemographic variables, number of chronic diseases, smoking status, and BMI, only fair/poor health remained higher for the restrictive impairment and moderate–severe obstruction groups. In contrast, prevalences of all poor HRQOL measures remained significantly higher for those who reported any chronic respiratory symptoms compared to asymptomatic individuals after adjusting for sociodemographic variables, number of chronic diseases, smoking status, and BMI. Prevalences of all poor HRQOL measures were also significantly higher for those who reported each of the chronic respiratory symptoms compared to individuals without the given symptom.

**Table 4:** Prevalence and likelihood of poor health-related quality of life measures by lung function category and presence of respiratory symptoms among US adults aged 40–79 - National Health and Nutrition Examination Survey, 2007-2010

	**%**^**a **^**(SE)**		**Unadjusted PR**^**a **^**(95%CI)**		**Adjusted PR**^**b **^**(95%CI)**		**Adjusted PR**^**c **^**(95%CI)**		**Adjusted PR**^**d **^**(95%CI)**	
**Fair or poor health**										
**Lung function category**										
Normal lung function	14.3	(1.0)	1.0	--	1.0	--	1.0	--	1.0	--
Restrictive impairment	33.1	(3.4)	2.3	(1.82-2.95)	1.6	(1.24-1.95)	1.5	(1.20-1.90)	1.5	(1.21-1.93)
Mild obstruction	11.1	(1.3)	0.8	(0.60-1.00)	1.0	(0.76-1.23)	1.0	(0.77-1.22)	1.0	(0.77-1.22)
Moderate-severe obstruction	26.0	(2.3)	1.8	(1.53-2.16)	1.6	(1.39-1.91)	1.5	(1.25-1.77)	1.5	(1.25-1.78)
**Chronic respiratory symptoms**										
Frequent cough	28.2	(2.1)	1.9	(1.57-2.26)	1.4	(1.17-1.77)			1.1	(0.87-1.47)
No frequent cough	15.0	(1.0)	1.0	--	1.0	--			1.0	--
Frequent phlegm	33.1	(2.4)	2.2	(1.87-2.63)	1.6	(1.27-1.94)			1.3	(1.00-1.74)
No frequent phlegm	14.9	(1.0)	1.0	--	1.0	--			1.0	--
Past year wheeze	29.8	(2.5)	2.1	(1.79-2.41)	1.4	(1.21-1.73)			1.3	(1.03-1.56)
No past year wheeze	14.4	(0.9)	1.0	--	1.0	--			1.0	--
Any	28.2	(1.8)	2.1	(1.87-2.44)	1.6	(1.38-1.80)	1.5	(1.30-1.71)		
None	13.2	(0.9)	1.0	--	1.0	--	1.0	--		
**Frequent physical distress**^**e**^										
**Lung function category**										
Normal lung function	10.2	(1.0)	1.0	--	1.0	--	1.0	--	1.0	--
Restrictive impairment	18.4	(2.3)	1.8	(1.28-2.54)	1.2	(0.81-1.78)	1.2	(0.77-1.74)	1.2	(0.78-1.76)
Mild obstruction	10.2	(1.5)	1.0	(0.70-1.45)	1.1	(0.76-1.56)	1.1	(0.76-1.55)	1.1	(0.76-1.52)
Moderate-severe obstruction	15.8	(1.9)	1.6	(1.20-2.01)	1.2	(0.94-1.62)	1.1	(0.83-1.47)	1.1	(0.79-1.41)
**Chronic respiratory symptoms**										
Frequent cough	21.7	(2.4)	2.2	(1.80-2.57)	1.6	(1.27-1.90)			1.1	(0.87-1.33)
No frequent cough	10.1	(0.7)	1.0	--	1.0	--			1.0	--
Frequent phlegm	26.8	(3.7)	2.7	(2.12-3.40)	1.9	(1.43-2.50)			1.6	(1.15-2.18)
No frequent phlegm	10.0	(0.7)	1.0	--	1.0	--			1.0	--
Past year wheeze	24.1	(2.3)	2.6	(2.07-3.18)	1.8	(1.43-2.22)			1.6	(1.21-2.08)
No past year wheeze	9.4	(0.8)	1.0	--	1.0	--			1.0	--
Any	20.5	(1.9)	2.3	(1.98-2.73)	1.6	(1.40-1.91)	1.6	(1.36-1.91)		
None	8.8	(0.7)	1.0	--	1.0	--	1.0	--		
**Frequent mental distress**^**f**^										
**Lung function category**										
Normal lung function	11.3	(0.7)	1.0	--	1.0	--	1.0	--	1.0	--
Restrictive impairment	15.7	(1.9)	1.4	(1.09-1.75)	1.2	(0.97-1.52)	1.2	(0.93-1.44)	1.2	(0.95-1.47)
Mild obstruction	9.6	(1.8)	0.8	(0.56-1.28)	1.0	(0.71-1.46)	1.0	(0.73-1.44)	1.0	(0.73-1.43)
Moderate-severe obstruction	12.5	(2.0)	1.1	(0.79-1.56)	1.0	(0.72-1.40)	0.9	(0.61-1.26)	0.9	(0.60-1.23)
**Chronic respiratory symptoms**										
Frequent cough	22.1	(2.3)	2.1	(1.71-2.67)	1.7	(1.33-2.21)			1.4	(0.99-1.96)
No frequent cough	10.3	(0.6)	1.0	--	1.0	--			1.0	--
Frequent phlegm	22.9	(2.7)	2.2	(1.70-2.73)	1.7	(1.32-2.27)			1.3	(0.91-1.84)
No frequent phlegm	10.6	(0.6)	1.0	--	1.0	--			1.0	--
Past year wheeze	21.9	(2.1)	2.2	(1.76-2.71)	1.7	(1.30-2.12)			1.5	(1.16-1.94)
No past year wheeze	10.0	(0.6)	1.0	--	1.0	--			1.0	--
Any	20.2	(1.7)	2.2	(1.78-2.68)	1.7	(1.40-2.18)	1.8	(1.40-2.22)		
None	9.3	(0.6)	1.0	--	1.0	--	1.0	--		
**Frequent activity limitation**^**g**^										
**Lung function category**										
Normal lung function	4.6	(0.6)	1.0	--	1.0	--	1.0	--	1.0	--
Restrictive impairment	9.4	(1.7)	2.0	(1.24-3.36)	1.3	(0.75-2.11)	1.2	(0.73-2.09)	1.3	(0.74-2.11)
Mild obstruction	4.7	(1.3)	1.0	(0.54-1.92)	1.1	(0.58-2.12)	1.1	(0.58-2.12)	1.1	(0.59-2.09)
Moderate-severe obstruction	8.8	(1.6)	1.9	(1.18-3.08)	1.4	(0.82-2.39)	1.3	(0.76-2.21)	1.3	(0.75-2.13)
**Chronic respiratory symptoms**										
Frequent cough	11.2	(1.4)	2.4	(1.93-3.03)	1.5	(1.13-1.88)			1.2	(0.81-1.71)
No frequent cough	4.7	(0.4)	1.0	--	1.0	--			1.0	--
Frequent phlegm	12.6	(1.4)	2.7	(2.06-3.45)	1.5	(1.14-2.10)			1.3	(0.83-1.93)
No frequent phlegm	4.7	(0.5)	1.0	--	1.0	--			1.0	--
Past year wheeze	11.6	(1.4)	2.6	(1.97-3.49)	1.5	(1.07-2.10)			1.3	(0.92-1.94)
No past year wheeze	4.4	(0.5)	1.0	--	1.0	--			1.0	--
Any	10.1	(1.1)	2.5	(1.91-3.19)	1.4	(1.09-1.92)	1.4	(1.07-1.85)		
None	4.1	(0.4)	1.0	--	1.0	--	1.0	--		

^a^ Unadjusted.

^b^ Prevalence ratio (PR) and 95% confidence interval (CI) obtained from multivariable logistic regression models that included either lung function category or presence of any respiratory symptoms, as well as sex, age, race/ethnicity, education, number of non-respiratory chronic illnesses, smoking status, and BMI.

^c^ Prevalence ratio (PR) and 95% confidence interval (CI) obtained from multivariable logistic regression model that included both lung function category and presence of any respiratory symptoms simultaneously and was adjusted for sex, age, race/ethnicity, education, number of non-respiratory chronic illnesses, smoking status, and BMI.

^d^ Prevalence ratio (PR) and 95% confidence interval (CI) obtained from multivariable logistic regression model that included both lung function category and each respiratory symptom simultaneously and was adjusted for sex, age, race/ethnicity, education, number of non-respiratory chronic illnesses, smoking status, and BMI.

^e^ Physical health not good ≥14 days during past 30 days.

^f^ Mental health not good ≥14 days during past 30 days.

^g^ Poor health limited usual activities ≥14 days during past 30 days.

In models including both lung function category and presence of respiratory symptoms (any versus none) simultaneously and adjusting for sociodemographic variables, number of chronic diseases, smoking status, and BMI, prevalence of fair/poor health remained significantly higher for participants with restrictive impairment (PR = 1.51 [1.20-1.90]) and moderate–severe obstruction (PR = 1.49 [1.25-1.77]) compared to those with normal lung function. In these fully adjusted models, all poor HRQOL measures remained significantly higher for participants who reported any respiratory symptoms compared to asymptomatic participants (fair/poor health: PR = 1.49 [1.30-1.71]; frequent physical distress: PR = 1.62 [1.36-1.91]; frequent mental distress: PR = 1.76 [1.40-2.22]; and frequent activity limitation: PR = 1.41 [1.07-1.85].) Since the association with HRQOL might differ by respiratory symptom, we also performed analysis using models that included septe variables for each symptom. When adjusting for the presence of the other respiratory symptoms, frequent cough was not associated with increased likelihood of any poor HRQOL measures. Frequent phlegm was associated with increased likelihood of fair/poor health and frequent physical distress. Past year wheeze was associated with increased likelihood of all poor HRQOL measures except frequent activity limitation.

## Discussion

In this study, we observed that restrictive and moderate–severe obstructive impairments were associated with several measures of poor HRQOL in unadjusted models. However, when we adjusted for chronic respiratory symptoms and other covariates in the analyses, most of the significant associations were attenuated or no longer significant, with the exception of fair/poor perceived health. On the other hand, all the HRQOL measures were significantly associated with any self-reported respiratory symptoms. These results suggest that although both pulmonary function and respiratory symptoms may contribute to poor HRQOL, respiratory symptoms may disproportionately impact more aspects of HRQOL than does pulmonary function. Aside from the mere presence of any respiratory symptoms, we also observed different associations between the specific symptoms and poor HRQOL measures. When adjusting for the presence of the other respiratory symptoms, frequent cough was no longer associated with increased likelihood of any poor HRQOL measures. Frequent phlegm was associated with increased likelihood of fair/poor health and frequent physical distress. Past year wheeze was associated with increased likelihood of all poor HRQOL measures except frequent activity limitation.

Some research has previously shown an association between pulmonary function and HRQOL among patients with COPD, asthma, and other respiratory diseases [3,9]. The relationship is generally stronger with the physical component of HRQOL than the mental or psychological component [3,7,9,11,12]. These findings are similar to our unadjusted results, where we observed a higher unadjusted prevalence of frequent physically unhealthy days among adults with restrictive or moderate–severe obstructive impairment, but no difference in prevalence of frequent mental distress based on pulmonary function category. However, once we adjusted for sociodemographic variables, chronic illnesses, smoking status, and BMI, we no longer observed a difference in frequent physically unhealthy days by pulmonary function category. These earlier studies also did not evaluate the association between respiratory symptoms and HRQOL septely from pulmonary function, and were therefore not able to ascertain how much poor HRQOL was attributable to impaired pulmonary function versus respiratory symptoms.

Our findings regarding respiratory symptoms are supported by several other studies. In an Australian adult population cohort, chronic cough was more common among those with severe psychological disturbance. This result persisted when the analyses were limited to individuals without identifiable respiratory disease and non-smokers. Compared to those without chronic cough at any time, adults reporting cough at follow-up were more likely to report more severe psychological disturbance, as well as poorer HRQOL (both physical and mental components) [19]. Another population study in Norway (N = 2306) showed that after adjustment for respiratory symptoms, the physical component scale score was significantly lower (worse) in individuals with severe COPD, and the mental component scale score was significantly higher (better) in individuals with COPD [3]. Similar to our study, both physical and mental components of HRQOL were negatively impacted by respiratory symptoms. The primary focus of that study was COPD and restrictive impairment and other obstructive impairment were not evaluated [3]. Among Swedish patients with asthma, symptom severity was associated with worse physical and mental well-being among women but not among men [20]. Other studies have found that respiratory symptoms were related to poorer HRQOL among COPD, asthma, and sarcoidosis patients, while pulmonary function was not [8,21]. Finally, in the European Community Respiratory Health Survey, individuals with respiratory symptoms had poorer physical and mental HRQOL even in the absence of asthma and COPD and after adjustment for bronchial hyperresponsiveness and FEV_1_[22].

Mild obstruction was not associated with any HRQOL measures in our study. Likewise, asymptomatic mild obstruction was not associated with poorer HRQOL in a Swiss cohort when compared to asymptomatic individuals with normal lung function. However, those with symptomatic mild obstruction not only had poorer HRQOL, they also had a faster decline in FEV_1_ and greater utilization of respiratory care [23]. This further emphasizes the importance of respiratory symptoms in overall health. In another study, individuals with mild obstruction were more likely to have depressive symptoms than adults without obstruction [4]. Unfortunately, data regarding respiratory symptoms was not included in that analysis. Since these individuals were selected for that study because they had been treated for COPD in the previous year, it is possible that they had more respiratory symptoms than if they had been selected from the general population.

The HRQOL questions used in this study were developed by CDC to provide generic measures of HRQOL rather than condition-specific measures in order to allow comparison across all population age groups and chronic conditions. They have been validated in general populations as well as numerous subpopulations and various conditions [18,24-26]. These measures are compble to subscales of other generic HRQOL instruments such as the Medical Outcomes Study Short Form-36 [27]. Although there is some literature on the association of pulmonary function and respiratory symptoms with HRQOL using condition-specific questionnaires such as the St. George’s Respiratory Questionnaire and the Airways Questionnaire 20, items used to assess the presence or severity of respiratory symptoms are overrepresented and therefore do not assess global measures of physical and mental health.

There are some limitations to this study. First, this was a cross-sectional study. Therefore, we cannot determine causality in the association between pulmonary function, respiratory symptoms, and HRQOL. Although adverse respiratory health likely leads to poor HRQOL, it is also possible that poor HRQOL could lead to worse management of symptoms or to behaviors that might be detrimental to respiratory health. Second, symptoms included in the questionnaire were limited. One important symptom that was not addressed was shortness of breath or dyspnea. The respiratory symptoms questions were also asked of a limited age group (aged ≥40 years). Therefore, our results may not be generalizable to younger populations.

Another limitation of this study lay in the large proportion of adults without spirometric results who were omitted from our analyses. There were 1224 adults with complete data on symptoms, HRQOL measures, and confounders, but missing spirometry data. Of these, 592 were safety exclusions, 315 underwent spirometric testing but of insufficient quality, and 317 did not undergo testing for other reasons such as participant refusal or time limitations. Compared to the study population, the safety exclusions were older, had more chronic illnesses, and were more likely to have a history of smoking. Similarly to the moderate-severe obstruction group, this group had a higher likelihood of reporting frequent cough, frequent phlegm, and past year wheeze. The persons who underwent spirometry but with insufficient quality results were also older, but were more likely to be of minority ethnic/racial groups, and to be never smokers. This group had a higher likelihood of reporting past year wheeze, but not frequent cough or frequent phlegm, than those with normal lung function. Participants excluded for other reasons were more predominantly female and had a higher proportion of minority ethnic/racial groups. This group also had a higher likelihood of reporting past year wheeze, but not frequent cough or frequent phlegm, than those with normal lung function. All three groups excluded from our analyses due to inadequate or no spirometry data had a higher likelihood of reporting fair/poor health, frequent physical distress, and frequent activity limitation (in fully adjusted models) than adults with normal lung function. The safety exclusion group also had a higher prevalence of frequent mental distress. Many of the participants in the safety exclusion group likely had severe lung function impairment, such as might require supplemental oxygen (see Table 1), but other problems may not have been associated with poor lung function at all.

## Conclusions

When respiratory symptoms were taken into account, pulmonary function was associated only with perceived fair/poor health. Respiratory symptoms, however, were strongly associated with all HRQOL measures. Most of this association appears to be attributable to frequent phlegm and past year wheeze. More than one quarter of the adults in this survey were identified to have some pulmonary dysfunction, while nearly one quarter of the adults in this survey reported at least one respiratory symptom including 20% of adults with normal lung function. Given the high prevalence of respiratory problems in the population, additional research is necessary to determine if diagnosis and treatment of pulmonary dysfunction or treatment of respiratory symptoms in the general population will improve HRQOL.

## Abbreviations

BMI: Body mass index; COPD: Chronic obstructive pulmonary disease; FAL: Frequent activity limitation (≥14 activity limitation days); FEV1: Forced expiratory volume in 1 second; FMD: Frequent mental distress (≥14 mentally unhealthy days); FPD: Frequent physical distress (≥14 physically unhealthy days); FVC: Forced vital capacity; HRQOL: Health-related quality of life; MEC: Mobile examination centers; NHANES: National Health and Nutrition Examination Survey; PR: Prevalence ratio.

## Competing interests

The authors declare that they have no competing interests.

## Authors’ contributions

AGW contributed to the study concept and design, acquisition of data, analysis and interpretation of data, drafting of the manuscript, critical revisions of the manuscript for important intellectual content, statistical analysis, and study supervision. ESF contributed to the analysis and interpretation of data, drafting of the manuscript, and critical revisions of the manuscript for important intellectual content. WWT contributed to the analysis and interpretation of data, drafting of the manuscript, and critical revisions of the manuscript for important intellectual content. KJG contributed to the analysis and interpretation of data, drafting of the manuscript, and critical revisions of the manuscript for important intellectual content. LRP contributed critical revisions of the manuscript for important intellectual content. JBC contributed to the drafting of the manuscript, critical revisions of the manuscript for important intellectual content and statistical analysis, and administrative, technical, or material support. All authors read and approved the final manuscript.

## Disclaimer

The findings and conclusions in this article are those of the authors and do not necessarily represent the official position of the Centers for Disease Control and Prevention.

## Pre-publication history

The pre-publication history for this paper can be accessed here:

http://www.biomedcentral.com/1471-2458/13/854/prepub
